# Content Development for a Multilevel Intervention to Operationalize Behavior Change Theory and Improve Parenting Behaviors for Physical Activity: Descriptive Study

**DOI:** 10.2196/73510

**Published:** 2025-08-26

**Authors:** Breanne C Wilhite, Erin Hennessy, Christina Economos, Jennifer Sacheck, Rachel Zive, Christine Odalen, Stephanie Herrick, Daniel P Hatfield

**Affiliations:** 1 The Gerald J. and Dorothy R. Friedman School of Nutrition Science and Policy, Tufts University, 150 Harrison Ave, Boston, MA, 02111, United States, 1 7815046421; 2 Department of Behavioral and Social Sciences, School of Public Health, Brown University, Providence, RI, United States; 3 Rising New York Road Runners, New York Road Runners, New York, NY, United States; 4 FHI 360, Washington, DC, United States

**Keywords:** school-based physical activity, multilevel intervention, behavior change theory, physical activity parenting, communication material design, theory-informed design

## Abstract

**Background:**

Theory-informed strategies for engaging parents in children’s physical activity (PA) promotion show promise. However, behavior-change interventions must become more rigorous in both their application of theory and the reporting of its use to continue to advance the field.

**Objective:**

This study aims to elucidate how 2 behavior change theories were used to develop parent communication materials in a 20-week communications campaign, nested within a multilevel (school-home) intervention to promote children’s PA. The innovation described in this study is derived from the Supporting Physical Literacy at School and Home (SPLASH) feasibility study (2021‐2022).

**Methods:**

A team of 7 experts, including graduate students, researchers, faculty, and child PA specialists, collaboratively designed the process used to develop the intervention content. With experience in theory-informed interventions and health-related communication campaigns, they held recurring meetings to refine the approach.

**Results:**

A four-step process was used to develop the theory-informed parent communication materials: (1) establish a theoretical foundation for communication materials (ie, social cognitive theory and self-determination theory) and conduct focus groups with the priority population; (2) identify and select PA parenting behaviors aligned with evidence and behavioral theories to form PA parenting objectives that advance children’s PA; (3) identify theoretical determinants of parent behavior change and outline methods for applying determinants to address PA parenting objectives; (4) operationalize theory-informed strategy and draft, review, and finalize materials. Parent communications were delivered through print materials and electronic channels, including email, text messages, Facebook (Meta Platforms, Inc), and activity videos.

**Conclusions:**

This descriptive study advances progress in the development of school-based PA promotion efforts seeking to incorporate parent engagement strategies by detailing how behavior-change theories can be operationalized to improve PA parenting behaviors. This methodology is valuable for others seeking to translate theoretical constructs into behavior-change communication messages.

## Introduction

Participating in regular and sufficient physical activity (PA) is an essential contributor to optimal growth and development in childhood and favorable physical and mental health throughout the life course [1,2]. In the United States, changes in lifestyle factors, the built environment, and opportunities for PA have resulted in declines in children’s PA, with disparities by gender, race, ethnicity, and family income becoming increasingly pervasive [3-6]. The development of strategies that can improve and maintain children’s PA is therefore a growing priority.

Based on the substantial amount of time school-aged children spend at school, the availability and accessibility of PA opportunities at school can have a significant impact on promoting children’s PA [7,8]. Reviews of school-based PA interventions have shown promise for improving children’s PA levels [9,10], with multicomponent, comprehensive school PA programs (ie, integration of multiple strategies during and outside school hours) potentially having the greatest impact on daily activity levels [11,12]. In addition, there is widespread belief that parent or caregiver (herein referred to as “parents,” which includes biological parents, other biological relatives who may act as caretakers, and nonbiological parents such as adoptive, foster, or stepparents) engagement in school-based PA promotion efforts is essential for providing the consistent opportunities and support children need to be physically active [10,13-17]. As such, developing school-based PA interventions that involve parents has become an area of growing attention.

Some commonly used parent engagement strategies in school-based interventions include establishing clear communication channels between school and home, providing opportunities for parents to build their knowledge, skills, and confidence for supporting their children’s behaviors, and engaging parents in activities at home to reinforce messages and practices taught at school [18]. However, the independent contribution that parent engagement strategies have on school-based intervention effectiveness, including child PA outcomes, is inconclusive [14,15,19,20]. A review by O’Connor et al [21] of school-based PA interventions with parental components found that inadequate and inconsistent reporting has made it difficult to interpret outcomes and draw conclusions on the most effective parent engagement strategies. Theory-based, empirically verified procedures are warranted to allow for a more systematic evaluation of such interventions’ impact on both parent and child behaviors and for their eventual widespread dissemination.

There has been progress in the broader behavior-change science field to develop systematic methods such as ontologies [22-24], taxonomies [25-27], methods for mapping interventions [28-30], and a theory coding scheme [31] that can be widely applied across the design, implementation, and evaluation phases of theory-based health behavior interventions [32,33]. However, continued inconsistencies in theory application and poor reporting of theory use make it difficult to ascertain and disseminate effective methods, limiting both the advancement and practical application of behavior-change strategies [34,35]. Furthermore, these systematic methods provide frameworks for intervention planning and design broadly, with none focusing on the operationalization of theory into intervention content, such as communication materials. This may help explain why studies of communication interventions seldom provide descriptions of how theory was applied to messaging and materials design, making it difficult to interpret study results, draw broader conclusions about whether given theories are useful and replicate methods.

This descriptive study aims to address these gaps in school-based intervention design by detailing the development of theory-informed communication materials intended to engage parents in children’s PA promotion efforts. More specifically, our work recounts how two behavior change theories were used to inform a 20-week multichannel communications campaign seeking to engage parents, nested within a school-based PA intervention (herein referred to as the “multilevel intervention”). This innovative descriptive study provides an example for operationalizing theory in communication messages for multilevel behavior change. These methods are of value to others seeking to be more rigorous in the application and reporting of theory use when designing parent engagement strategies to promote children’s PA.

## Methods

### The Supporting Physical Literacy at School and Home Study

This work was conducted in partnership with New York Road Runners (NYRR). NYRR is the world’s premier community running organization with a mission to help and inspire people through running. They have disseminated youth running and PA programming since 1999. In 2017, NYRR released a new running-based youth program, Rising New York Road Runners (RNYRR), that can be incorporated into before-, during-, and after-school PA opportunities. RNYRR programming focuses on enjoyable, inclusive, developmentally appropriate games and activities that sequentially build motor skills in one or more domains (eg, locomotion, balance, and object control) to improve children’s physical literacy (PL), defined as their confidence, competence, and motivation to be physically active for life [36,37].

The innovation described in this descriptive study is derived from the multilevel intervention, the Supporting Physical Literacy at School and Home (SPLASH) feasibility study (NCT05887583). Implemented during the 2021‐2022 school year, SPLASH was designed to support RNYRR’s existing school-based PA program by developing and delivering strategies to engage families in children’s PA beyond school as well.

The aims of the SPLASH study included: (1) the development of a theory-informed home-based communications component, and (2) an assessment of implementation feasibility and acceptability in the target population. Aim 1 is the focus of this descriptive study, and results from aim 2 are forthcoming.

### Key Programmatic Elements

A 4-step process was used to develop the theory-informed parent communications. Each step in the process included several activities that were informed by theory and existing evidence. The outputs generated from the activities were then integrated into the subsequent step. The entire 4-step process serves as an example of designing theory-informed communication materials intended to engage parents in children’s PA. Table 1 outlines the steps, activities, and outputs.

**Table 1.** Development process of theory-informed communication materials.

Step	Activity	Output
Step 1: formative research		
Theoretical foundations	Aligned theoretical foundations for communication materials with overall multilevel intervention aims.	Theoretical basis established for communication materials.
Focus groups	Conducted focus groups with the priority population.	Summarized findings to tailor communication content.
Step 2: behavior change objectives	Conducted a literature review to identify and select PA parenting behaviors aligned with existing evidence and theoretical basis.	Parenting objectives are formed to advance children’s PA^a^.
Step 3: theory-informed strategy	Identified theoretical determinants of parental behavior change and outlined the application of determinants.	A strategy created to address PA parenting objectives.
Step 4: operationalization	Selected communication channels and drafted, reviewed, and edited materials to operationalize the theory-informed strategy.	Communication materials finalized.

a PA: physical activity.

Step 1 (formative research) took place during the grant proposal design phase in 2018‐2019. Steps 2‐4 took place from fall 2019 to spring 2021, with implementation of the multilevel intervention beginning in fall 2021.

The process included a planning and design team of 7 members, consisting of graduate students, research managers, research scientists, and faculty members from leading universities in nutrition, PA, and behavior change, and child PA specialists from RNYRR. The process was collaborative and iterative, consisting of recurring team meetings to review and discuss progress. Many team members had previously worked together on similar school- and community-based projects, including studies testing theory-informed approaches to promote child PA and improve health-related parenting practices [16,38-41]. Collectively, our team consisted of PA experts with experience in applying behavior change theories to intervention design and in developing and implementing communication campaigns to improve children’s health behaviors.

### Ethical Considerations

This study was reviewed and approved by the Social, Behavioral, and Educational Research Institutional Review Board at Tufts University (approval no 1812027; January 2019). All procedures were conducted following the ethical standards of the institutional research committee and the Declaration of Helsinki and later amendments. Informed consent was obtained from all participants before data collection. All data was deidentified and participants recieved a $50 Visa prepaid debit card for their time.

## Results

### Step 1: Formative Research

#### Theoretical Foundations

The theoretical basis for the communications campaign was selected during the grant proposal design phase in 2018. The National Institutes of Health Funding Opportunity Announcement (PAR-14‐321) encouraged applications for “Phased Innovation (R21/R33) grant awards to support highly innovative research aimed at developing multilevel interventions that will increase health-enhancing PA: (1) in persons or groups who can benefit from such activity, and (2) that can be made scalable and sustainable for broad use across the nation.” In response to this funding announcement, the authors partnered with NYRR to expand upon RNYRR’s school-based PA program. We proposed developing strategies that would engage families in children’s PA learning outside of school time to complement and reinforce the in-school programming. As the RNYRR school curriculum was developed with a focus on increasing children’s PL, we sought to draw upon theoretical frameworks that would complement PL tenets when designing the family communication materials.

The concept of PL has been defined in several ways; for the multilevel intervention, we adopted the definition from the Aspen Institute’s landmark 2015 report: “physical literacy is the ability (competence), confidence, and desire (motivation) to be physically active for life [42].” According to that report, ability or competence includes fundamental movement skills, which provide the foundation for a range of PA; confidence is one’s belief in their ability to participate in sports or other physical activities; and desire or motivation includes intrinsic enthusiasm for PA.

Social cognitive theory (SCT) and self-determination theory (SDT) are two theoretical frameworks that complement PL concepts. For example, SCT constructs of self-efficacy and behavioral skills are similar to the confidence and competence components of PL [43], and SDT centers on motivational states and therefore aligns with the motivational dimension of PL [44]. Furthermore, SCT and SDT are commonly used in the design of interventions to target the determinants of children’s PA, aiming to improve the promotion and maintenance of PA across the lifecycle [43,45-47]; PL-based approaches focus on not just eliciting near-term PA but also enabling children to be “active for life [42].” Given the theoretical and empirical evidence for SCT- and SDT-based strategies in promoting children’s PA, the alignment between these theories and PL concepts, and the study team members’ prior experience with the application of both theories, we selected SCT and SDT as the theoretical basis for our communication materials. Table 2 summarizes the relevant literature for SCT and SDT in children’s PA behavior change.

**Table 2.** Summary of social cognitive theory and self-determination theory supporting evidence for children’s physical activity (PA) behavior change.

Overview of theory	Behavior change mechanisms	Evidence for children’s PA	Long-term maintenance of behavior changes	Application to intervention design
Social cognitive theory (SCT)				
Learning occurs in a social context through dynamic, reciprocal interactions between cognitive, behavioral, and environmental factors [48].	Behavior change is bidirectional and shaped by interactions between individuals and their environment, including others [49].	Observational learning, goal setting, and reinforcement are key to effective family-based interventions for increasing children’s PA [50].	For long-term maintenance of behavior, SCT emphasizes the importance of a supportive environment [45].	SCT effectively supports school-based interventions by engaging parents and extending children’s PA support across school and home environments.
Self-determination theory (SDT)				
Three innate and universal human needs underlie growth: autonomy, competence, and relatedness [51].	Behavior change relies on motivation and occurs when an individual’s psychological and physiological needs are met [51].	A motivational climate that fosters effort, personal growth, and decision-making opportunities significantly improves children’s PA outcomes [47].	For long-term maintenance of behavior, SDT emphasizes the importance of sustained intrinsic motivation for the behavior [45].	SDT is ideal for PA interventions that enhance children’s intrinsic motivation by fostering enjoyment and interest [52].

Though both theories help to explain the determinants of and conditions necessary for promotion and maintenance of a behavior, integrating components of the two may provide a more comprehensive understanding and improve the overall effectiveness of an intervention [9]. The integration of SCT and SDT has been a promising area of interest in designing and implementing school-based PA interventions [53,54]. Underlying these studies is the concept that the three basic human needs outlined in SDT (ie, autonomy, competence, and relatedness) can be satisfied or inhibited by the cognitive, behavioral, and environmental factors outlined in the SCT [55]. For example, social environmental factors, such as support for PA (ie, encouraging children’s choices, options, and opportunities to participate in PA) from multiple sources, may positively influence students’ motivation for and long-term engagement in PA [56,57]. Conversely, autonomous motivation for PA can positively influence factors such as attitudes toward PA, thereby influencing PA intention [58].

While the overall behavioral objective of the multilevel intervention was to promote children’s PA, the intention of our theory-informed communication materials was to engage parents and improve their PA parenting behaviors. By operationalizing theoretical constructs into the materials, we hoped to target the SCT and SDT determinants of PA parenting behaviors, thereby influencing the PA parenting behaviors. The improvement in PA parenting behaviors could then enhance the SCT and SDT determinants of their children’s PA and promote their children’s PA behaviors.

**Figure 1.**
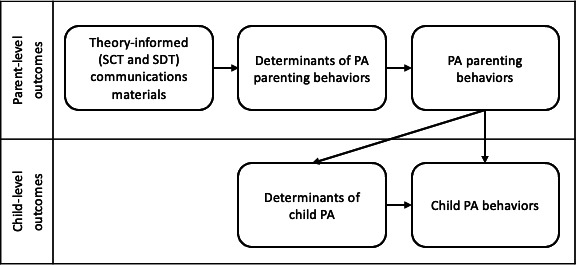
Promoting children’s PA through theory-informed communication materials targeting PA parenting behaviors. PA: physical activity; SCT: social cognitive theory; SDT: self-determination theory.

Figure 1 depicts the relationship between the theory-informed (ie, SCT and SDT) communication materials and parental- and child-level behavioral outcomes. By operationalizing theoretical constructs into the materials, we aimed to target the SCT and SDT determinants of PA parenting behaviors, thereby influencing the PA parenting behaviors. Improvement in PA parenting behaviors could then enhance the SCT and SDT determinants of children’s PA and promote their PA behaviors.

#### Focus Groups

Focus groups were conducted with parents from the priority population to inform the overall development of the theory-informed parent communications. These focus groups explored parental knowledge and attitudes around child PA, parental perceptions of their children’s PA engagement, perceived barriers and facilitators to supporting their children’s PA, and current or preferred school-family communication channels.

Eight focus groups were conducted with parents of children in grades 3‐5 in both the NYC and Boston areas between April and June 2019. Of the parents who participated in a focus group (n=69), 30% and 48% identified as non-Hispanic Black and Hispanic, respectively, and 89% identified as mothers. Results were coded by 3 study team members and organized by major themes. Primary results used in developing the materials include the following: (1) parents saw their children as lacking sufficient PA and viewed themselves as primary motivators, facilitators, and role models. Ensuring adequate PA for their children was a priority for them, (2) parents valued PE for teaching their children PA skills but wanted more school communication and involvement in PA opportunities. They preferred diverse communication methods with no single preferred frequency, and (3) parents wanted to engage in family PA but faced barriers such as time, energy, motivation, and space. They sought activity ideas aligned with PE lessons, adaptable for the whole family, and feasible in a limited space.

These findings contributed to a clearer understanding of the challenges faced by parents in our priority population to support or engage in PA with their children and highlighted potential opportunities to better connect families with schools. These findings were incorporated throughout the communication design process to ensure the final materials were responsive to the needs and preferences of the priority population.

### Step 2: Behavior Change Objectives

To engage parents to promote children’s PA, we needed to first identify priority PA parenting behaviors and the determinants of those PA parenting behaviors within the context of our theoretical basis. Reviewing the literature through the lens of SCT and SDT allowed us to understand how parents could both serve as an important socioenvironmental influence in the context of SCT and help satisfy needs in the context of SDT [59-61]. Our goal in step 2 was to (1) categorize the most influential parenting behaviors for promoting children’s PA based on our literature review of existing evidence and (2) ensure the PA parenting behaviors aligned with the formative research conducted in Steps 1a and 1b. Collectively, step 2 details how our PA parenting objectives relate to the theoretical determinants of children’s PA.

Our review of the parenting literature was conducted in the summer of 2019 by 3 study team members and supported by a Tufts University librarian. Web of Science, PubMed, and Ovid databases were used with a broad search strategy across all years of publication. Example search terms, informed by similar reviews, included terms related to parents (parent* OR famil* OR caregiver* OR family carer*), children (pediatric* OR child* OR kid* OR adolescen*), parent engagement (involv* OR participat* OR engage* OR outreach OR recruit* OR retain* OR retention*), intervention type (community-linked OR community-based OR school* OR teacher* OR student* OR classroom*), and child behavioral outcomes (exercis* OR recreation* OR fitness* OR physical activit*).

After reviewing 423 abstracts and 27 full-text papers, 3 categories of parental behaviors emerged as being supportive of children’s PA behavior change: modeling of PA, parental support of child PA, and parental encouragement for child PA [50,62-64]. Termed collectively as “PA parenting practices,” these behaviors are influenced by parents’ own PA attributes and perceptions. For example, parents with a positive perception of PA, who engage in, value, and enjoy PA, tend to have a high self-efficacy to promote their child’s PA. In addition, parents who perceive that their child enjoys and is competent in PA are more likely to adopt responsive and structured PA parenting practices [65,66]. Responsive and structured PA parenting practices can, in turn, indirectly influence children’s PA by enhancing children’s modifiable PA attributes, such as their perceived competence, self-efficacy, and motivation for PA [66-68]. Some PA parenting practices, such as supportive behaviors, are associated with a direct increase in children’s PA levels [50,64]. Therefore, by aiming to improve PA parenting practices through our materials, our intervention aimed to target parents as potential mediators of their child’s PA behaviors [16,17].

The final parental behavior change objectives, with a brief description of specific behaviors, for our communications campaign were as follows. First, parents will serve as role models for their children’s PA. Parents will model enjoyable and relevant PA behaviors individually and with their children. Second, parents will provide instrumental and informational support, including the provision of choices, for their children’s PA. Specifically, (2a) parents will provide choices and descriptions of activities for their children to select from, and (2b) parents will promote a sense of familial connection around PA. Third, parents will provide praise and encouragement for their children’s PA. This includes (3a) giving frequent, direct, specific, and positive feedback and encouragement regarding some aspect of their children’s performance, and (3b) encouraging the use of process goals that allow for success and are achievable.

Table 3 exemplifies the relationships between the 3 parental behavior change objectives and the previously established theoretical basis for children’s PA behavior change.

**Table 3.** Relationships between physical activity (PA) parenting objectives and theoretical constructs of children’s PA.

PA parenting behavioral objective	Theoretical basis for children’s behavior change (related theory)
Parents serve as role models for their children’s PA	Observing parents (as influential role models) successfully performing the behavior can improve children’s self-efficacy (SCT^a^) and promote feelings of relatedness (SDT^b^) and competence (SDT), while shifting outcome expectations (SCT).
Parents will provide instrumental and informational support, including provision of choices, for their children’s PA	Providing activity choices and a rationale for all prescribed activities supports the development of autonomy (SDT) and knowledge (SCT) of engaging in PA for the children. Development of familial support (SCT) for the children can encourage feelings of relatedness (SDT).
Parents will provide praise and encouragement for their children’s PA	Positive feedback from parents supports the development of self-efficacy (SCT) and perceptions of competence (SDT) in the children. Process goals that are moderate and focus attention on the task (rather than performance relative to others) support autonomy (SDT), perceived competence (SDT), and self-efficacy (SCT).

a SCT: social cognitive theory.

b SDT: self-determination theory.

### Step 3: Theory-Informed Strategy

To target the parental objectives in our communication materials, we needed to (1) identify the theoretical determinants of the PA parenting behaviors we wished to change and (2) outline the methods for addressing the determinants in a communication campaign. Collectively, step 3 can be thought of as the theory-informed strategy for advancing our PA parenting objectives. Here, our definition of “theory-informed” is that the strategy for operationalization is informed by behavioral and social science theories [28]. Our theoretical basis provides evidence for why we anticipate our strategy will result in the desired parental behavior change.

Within the theoretical basis, we identified the determinants of the PA parenting behaviors we aimed to influence in our communications campaign. This process included a combination of literature and expert review from our team. After identifying the SCT or SDT constructs indicative of the parenting behavior, we then described the role of the theoretical determinant in advancing the behavioral objective. All constructs were contextualized based on their reciprocal relationships with one another. After determining the constructs predictive of the desired behavior change, we sought to outline the application of the constructs, or the methods for operationalizing the determinants into the communication messages. Outlining the methods for operationalizing the theoretical constructs required careful consideration for how to address the determinants through communication messages.

Table 4 shows how the PA parenting objectives were linked to methods via the behavioral determinants. The methods were applied by operationalizing the theoretical constructs into the communication materials in step 4.

**Table 4.** Theory-informed strategy for achieving physical activity (PA) parenting behavioral objectives.

Objective	Theoretical determinants of parent behavior change	Methods for addressing determinants
Parents will serve as role models for their children’s PA	1. Improving knowledge (SCT^a^) about the role parents play in children’s PA can influence realistic outcome expectations (SCT) for parents and encourage them to be role models. 2. Giving families opportunities (SCT) for observational learning (SCT) can enhance parents’ behavioral skills (SCT) for role modeling behaviors. 3. Providing enactive mastery experiences can enhance parents’ self-efficacy (SCT) and perceived competence (SDT^b^) for role modeling behaviors.	1. Parents receive information on the importance of their role in their children’s PA. 2. Parents are given instructions and ideas for how to model activities with their children. 3. Parents are encouraged to lead their children’s PA by modeling activities with supportive and accessible prompts.
Support for their children’s PA	1. Sharing new and enjoyable opportunities (SCT) for family activities can enhance parents’ autonomy (SDT) for providing instrumental support for their children’s PA. 2. Giving parents suggestions for how to reduce barriers (SCT) to PA can enhance parents’ self-efficacy (SCT) for providing informational support and provision of choices for their children’s PA.	1. Parents are given activity choices, with a clear description of each, and prompted to do the activities with their children. 2. Parents are provided with guidance on how to overcome common barriers to engaging in PA with their children, such as space or time constraints.
Parents will provide praise and encouragement for their children’s PA	1. Providing information and examples about the role of encouragement and praise in their children’s PA can improve parents’ knowledge (SCT) and self-efficacy (SCT) for encouraging and praising their children’s PA. 2. The act of self-monitoring and setting goals (SCT) or intentions (SCT) as a family and reinforcing (SCT) them every week throughout the program can improve parents’ perceived competence (SDT) for encouraging and praising their children’s PA.	1. Parents receive background information and user-friendly prompts to give frequent, specific, and positive praise for their children’s PA behaviors. 2. Materials reference and discuss the use of process goals for parents to use to encourage their children’s PA.

a SCT: social cognitive theory.

b SDT: self-determination theory.

### Step 4: Operationalization

In the final design step, the theory-informed messages were written and fully integrated into communication materials. Communication channels included emails, text messages, Facebook posts, and newsletters. The process for drafting and editing the messages was iterative and involved the entire research team.

**Figure 2.**
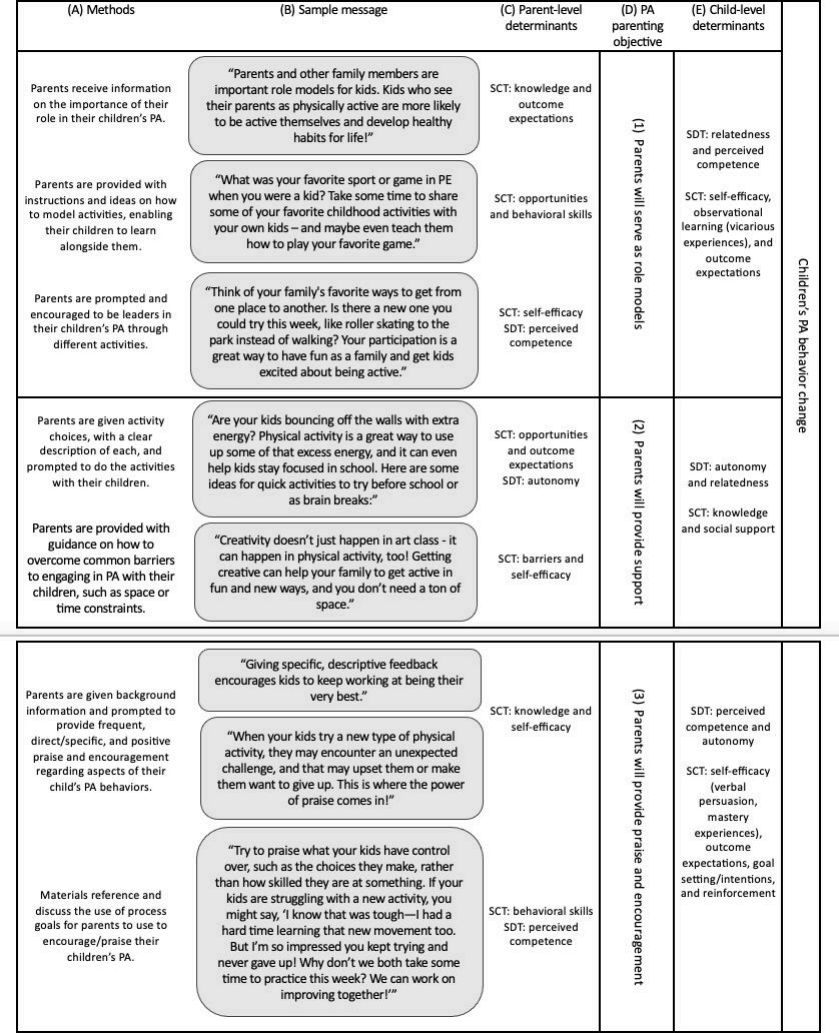
Operationalization of theory-informed strategy for achieving physical activity ( PA ) parenting behavioral objectives. SCT: social cognitive theory; SDT: self-determination theory; PA: physical activity.

Figure 2 includes sample messages exemplifying how we operationalized our strategy to achieve our PA parenting behavioral objectives. Each sample message is a manifestation of one or more methods (Figure 2A). In operationalizing our strategy, we can target key parent-level determinants (Figure 2C) that influence our PA parenting behaviors (Figure 2D). The PA parenting objectives are linked to changes in children’s PA behavior via the child-level determinants (Figure 2E).

Two study team members worked together to write the first draft of the family communication messages. One Tufts team member served as the behavior change expert, ensuring the theoretical constructs, methods, and parameters identified in step 3 were incorporated into the content; the other RNYRR team member served as the curriculum expert, ensuring the school-based curriculum was aligned with the family content. Both team members carefully deconstructed each message and mapped theoretical constructs to the messages. A grid was created to keep track of construct operationalization, ensuring intentionality. Input was obtained from other team members over multiple rounds and edits. Careful consideration was taken to ensure the content was appropriate for the target audience’s sociodemographic, cultural, and educational backgrounds. Messages were refined and final edits were presented to and agreed upon by all team members. Final materials were translated into Spanish for the study’s target audience.

Email served as the primary communication channel, with the other modes of communication written to reinforce that channel. All materials were aligned with the program theme, Rising Together, emphasizing the importance of being active as a family. Final material design was overseen by RNYRR to ensure consistency in appearance. When families signed up to receive the materials, they had the option to sign up for emails only, text messages only, or both emails and texts. To achieve equity in access to materials, all email communications were printed and delivered to participating schools to be placed into children’s backpacks on a weekly basis. Print-based emails contained QR codes that could be scanned to access the electronic content. The 20-week communications campaign and multilevel program were pilot tested in 6 NYC elementary schools during the 2021‐2022 school year.

## Discussion

### Principal Findings

This descriptive study details a 4-step process used to operationalize behavior change theory in a multichannel communications campaign, nested within a multilevel PA intervention. We describe systematic procedures that can be used in designing behavior change communication messages seeking to improve PA parenting behaviors and promote children’s PA.

The ability of parent engagement strategies to effectively engage parents and promote children’s PA in school-based interventions remains inconclusive [18]. We provide a practical 4-step process using commonly cited strategies that can be applied and empirically evaluated for their independent impact on school-based intervention effectiveness [14,50]. As Bandura [69] noted, public health campaigns are important for getting people to adopt health behaviors. However, health communications foster adoption of health behaviors to the extent that they raise individuals’ self-efficacy. Health communications must therefore be designed to enable people to build the necessary skills and self-beliefs needed to change their behaviors. Given the intentionality and feasibility of our communications to engage parents and impact parental-level determinants and behaviors, future applications of our methods can begin to advance understanding of the isolated impact of parent engagement strategies on child-level outcomes.

### Comparison With Prior Work

To our knowledge, this 4-step design process is innovative within multilevel behavior change interventions. Similar strategies have been used in designing interventions for various behaviors, including medication adherence [70], hand hygiene compliance [71], and occupational sedentary behavior [33], and in various contexts, including tailored feedback during a weight loss intervention [72] and a garden-enhanced nutrition curriculum [73]. However, only one of these interventions [74] addresses the complexity of promoting behavior change through an additional level of influence, as we aim to do in influencing PA parenting practices to promote children’s PA. This additional level of behavior change makes our approach valuable for others seeking translatable methods for applying theory-informed strategies to multilevel behavior change, such as in efforts to promote children’s PA.

Current frameworks for theory application do not include specific methods for designing health communications as part of intervention design [29,30,75]. While the theoretical evidence includes guides for translating theory-based methods into practical applications, these frameworks fall short of providing advice for operationalizing theoretical determinants into behavior change communication messages. Furthermore, while the empirical evidence provides examples of how theoretical constructs were translated into behavior change methods [70] or intervention activities and components [33,73,74], none have been specific to the translation of theory for the content of communications. This descriptive study addresses gaps in current intervention design frameworks and studies and complements their use by providing practical advice and examples for operationalizing theory into communication messages. The methods used in this descriptive study may, therefore, be used by interventionists in congruence with current frameworks to create communications that can be incorporated into multilevel behavior change interventions, which have been recommended for increasing children’s PA [13].

While there are many functions and benefits of theory application during the design, implementation, and evaluation stages of interventions [49,75-77], increased effectiveness is often cited as the primary reason for the use of theory in behavior change intervention design [78]. This argument may hold for theory-informed approaches to promote children’s PA [46,49,50], despite inconsistent conclusions on whether the use of theory improves intervention effectiveness [15]. Prior studies have noted that the variable findings on the effectiveness of behavior change interventions, including those for children’s PA, could be explained by “methodological and reporting issues at study and review level,” including issues with how theory is applied during the intervention design process [78]. Michie and Prestwich [31], for example, point out that “where a theoretical base for an intervention is stated, there is seldom reference to a method describing how the theory informed the design of the intervention.” By providing a detailed example and replicable methods for applying theory to communication materials, one of the crucial steps in the intervention life cycle, we believe our study to be of benefit to others attempting to be more rigorous in both their application of theory and reporting of its use in PA interventions.

This descriptive study’s description of the operationalization of theory in our communication materials will provide important contextual information for interpreting future results of the communications campaign. The feasibility of implementing the communications campaign and the acceptability of the materials for the target population will also be considered in a pilot study, and adjustments to the materials will be made. The adapted communications will be implemented in a randomized controlled trial. Findings from the trial will be used to understand if our theory-informed communications effectively engage parents, thereby influencing their determinants of PA and PA parenting behaviors, while examining the overall impact of the intervention on children’s PA determinants and PA behaviors. After a second phase of adjustment, including any adjustments to the methods outlined in this study, the communications may be considered for dissemination across the NYRR network of schools. Given the diversity and reach of the schools and the continuous feedback solicited by the program, the materials may continue to be adapted to fit local contexts and populations. The present descriptive study, together with future research plans, will therefore jointly advance the broader field of childhood PA research and practice.

### Limitations

Given the lack of examples in the literature, our descriptive study is not without constraints. For example, when operationalizing the theory-informed strategy from step 3 into communication messages in step 4, we lacked explicit guidance on any methodological parameters that should be considered. As previously discussed by Kok et al [30], the effectiveness of a behavior change method will depend on whether the parameters for the method are satisfied. In other words, a theory-informed strategy requires more than outlining what constructs to target and what methods to use to operationalize the constructs; it requires a consideration for the “theoretical parameters under which the theoretical process is effective or not” [30,79]. An example in the context of our descriptive study would be that improving parental self-efficacy requires a series of reinforcing messages, as opposed to a single message [49]. Parameters for operationalizing theory are not clearly defined for the application of parenting behavior change. Empirical evidence of what conditions were used when operationalizing theory for parenting behaviors will be required to determine the relevant parameters for effectiveness.

Participation of the target population is important for establishing the most relevant theoretical determinants of a behavior or for understanding how theory-informed strategies should be applied in the design process [29]. Our formative research with the priority population, including focus groups with parents, focused on topics such as communication channel preference and the feasibility of engaging parents through communication materials but did not include an explicit theoretical focus. Additional formative research informed by SCT and SDT may have enabled further tailoring in how we operationalized theory in our communication messages.

### Conclusions

This descriptive study advances progress in the development of school-based PA promotion efforts seeking to incorporate parent engagement strategies by detailing the operationalization of theory into communication messages. Future application of our 4-step process can be used to develop theory-informed communication materials that aim to target PA parenting behaviors.

The methodology detailed in this descriptive study is valuable for others seeking to translate theoretical constructs into behavior-change communication messages. This work provides an example and draws attention to the role of rigorous theory application and reporting in developing communications that support children’s PA behavior change as part of overall behavior-change intervention design.

## Abbreviations

NYRR: New York Road Runners
PA: physical activity
PL: physical literacy
RNYRR: Rising New York Road Runners
SCT: social cognitive theory
SDT: self-determination theory
SPLASH: Supporting Physical Literacy at School and Home
